# Supplementary material for: Nucleosomes influence multiple steps during replication initiation
Source: eLife. 2017 Mar 21;6:e22512. doi: 10.7554/eLife.22512 (PMC5400510; doi:10.7554/eLife.22512)
Supplement: Supplementary file 1. — DOI: http://dx.doi.org/10.7554/eLife.22512.026 [file elife-22512-supp1.docx]

**Supplementary File 1A**

**Yeast strains used in this study.**

| **Yeast Strain** | **Description** | **Reference** |
| --- | --- | --- |
| TAP-Ioc3 | TAP-tagged Ioc3 to purify ISW1a | Open biosystems |
| TAP-Ioc2 | TAP-tagged Ioc2 to purify ISW1b | Open biosystems |
| TAP-Ino80 | TAP-tagged Ino80 to purify INO80-C | Open biosystems |
| TAP-Chd1 | TAP-tagged Chd1 to purify Chd1 | Open biosystems |
| TAP-Rsc2 | TAP-tagged Rsc2 to purify RSC | Open biosystems |
| TAP-Swi2 | TAP-tagged Swi2 to purify SWI/SNF | Open biosystems |
| YTT480 | *MATa ade2-1 can1–100 his3–11,15 leu2–3,112 trp1-1 ura3-1 RAD5*+ *pep4*::*HIS3 ISW2-2FLAG* | Zofall et al., 2006 |
| ySK100 | *MATa ade2-1 ura3-1 his3-11,15 can1-100 bar1::HisG orc1∆::HisG pep4∆::KanMX*  *LEU2::ORC1*  *TRP1::p404-GAL1-10-ORC3,4 LYS2::plys2-GAL1-10-ORC2,5 HIS3::p403-GAL1-10-1xFlagORC1,6* | Kang et al., 2014 |
| yST144 | *MATa ade2-1 trp1-1 leu2-3,112 his3-11,15 ura3-1 can1-100 bar1∆::HisG lys2∆::HisG pep4∆*  *HIS3::pSKM004 (GAL1,10-MCM2, Flag-MCM3) URA3::pALS1 (GAL1,10-Cdt1, GAL4)*  *LYS2::pSKM002 (GAL1,10-MCM4, MCM5) TRP1::pSKM003 (GAL1,10-MCM6, MCM7)* | Ticau *et al*., 2015 |
| yRH146 | *ade2-1 trp1-1 leu2-3,112 his3-11,15 ura3-1 can1-100 bar1∆ lys2∆::HisG pep4∆::KanMX*  *LEU2::pAS584-DBF4-Flag, CDC7* | Heller *et al.,* 2011 |
| ySK119 | *URA3::pRS306 Gal1,10 deltaN95 CLB5-Flag CDC28-6XHis* | This study |
| ySK123 | *HIS3::pRS403 Gal1,10 SLD7-VSVG*  *LEU2::pRS405 Gal1,10 SLD3-deltaN104-3xFlag* | This study |
| ySK127 | \| *MATa pep4::unmarked bar1::hisG*  *LEU2::pGAL1/10-3xFLAG-3C-SLD2* \| \| --- \| | This study |
| yRH154 | *ade2-1 trp1-1 leu2-3,112 his3-11,15 ura3-1 can1-100 bar1∆ lys2∆::HisG pep4::KanMX LEU2::pAS584-Gal-DPB11-Flag* | This study |
| yMM016 | *MATa pep4∆ bar1∆::hisG LEU2::pGAL1/10-CDC45-FLAG* | This study |
| yMH28 | *MATa pep4::unmarked bar1::hisG TRP1::pGAL1/10-POL2-3C-5xFLAG URA3::pGAL1/10-DPB3+DPB4-3C-His + pGAL1/10-DPB2-3C-FLAG* | This study |
| yAS3 | *ade2-1 trp1-1 leu2-3,112 his3-11,15 ura3-1 can1-100 bar1∆ lys2∆::HisG pep4∆ TRP1::pBLM10(GAL1,10-PRI1,PRI2) HIS3::pBLM20(GAL1,10-POL1,POL12)*  *Pol12-PP-2FLAG* | This study |
| pMLy049 | *ade2-1 trp1-1 leu2-3,112 his3-11,15 ura3-1 can1-100 bar1∆ lys2::HisG pep4∆*  *LEU2::MCM10-FLAG* | This study |
| yMW004 | *ade2-1 trp1-1 leu2-3,112 his3-11,15 ura3-1 can1-100 lys2∆::HisG pep4∆::Hph*  *cdc7-1 LEU2:: pRH113* (*GAL1,10 Cdc45-3HA Dpb11*)  *LYS2::pRS307-GAL1, 10 SLD2 SLD3*  *URA3::pRS306-GAL1,10 CDC28 CLB5* | This study |
| yIA001 | *ade2-1 trp1-1 leu2-3,112 his3-11,15 ura3-1 can1-100 lys2∆::HisG pep4∆::Hph*  *HIS3::* *pJF17 (GAL1,10-ORC3, ORC4)*  *TRP1::* *pJF18 (GAL1,10-ORC5, ORC6)*  *URA3::* *pIA01 (GAL1,10-CBP-TEV-ORC1∆BAH, ORC6)* | This study |
| yIA002 | *MATa ade2-1 trp1-1 leu2-3,112 his3-11,15 ura3-1 can1-100 bar1∆::HisG lys2∆::HisG pep4∆*  *HIS3::pIA02 (GAL1,10-MCM2-2A, Flag-MCM3) URA3::pALS1 (GAL1,10-Cdt1, GAL4)*  *LYS2::pSKM002 (GAL1,10-MCM4, MCM5) TRP1::pSKM003 (GAL1,10-MCM6, MCM7)* | This study |
