# Supplementary material for: Nucleosomes influence multiple steps during replication initiation
Source: eLife. 2017 Mar 21;6:e22512. doi: 10.7554/eLife.22512 (PMC5400510; doi:10.7554/eLife.22512)
Supplement: Supplementary file 2. — DOI: http://dx.doi.org/10.7554/eLife.22512.027 [file elife-22512-supp2.docx]

**Supplementary File 1B**

**Plasmids used in this study.**

| **Plasmids** | **Description** | **Source** |
| --- | --- | --- |
| pET3aTr yH2A | *yH2A cloned into pET3aTr* | Vary *et al*., 2004 |
| pET3aTr yH2B | *yH2B cloned into pET3aTr* | Vary *et al*., 2004 |
| pET3aTr yH3 | *yH3 cloned into pET3aTr* | Vary *et al*., 2004 |
| pET3aTr yH4 | *yH4 cloned into pET3aTr* | Vary *et al*., 2004 |
| pET15b-hNap1 | *hNap1-His_6_ cloned into pET15b* | Eaton *et al*., 2010 |
| p11d-tscRPA-30MxeHis6 | Expression construct for yRPA1, yRPA2, yRPA3 | Gibb *et al*., 2014 |
| pSKM03 | *pGEX-Flag-Cdc6* | Kang *et al*., 2014 |
| pMH07 | *POL2* with *6Gly-PP-5xFLAG* | This Study |
| pMH01 | *DPB2* with *6Gly-PP-FLAG* to | This Study |
| pSKM002 | *pRS307 (GAL1,10-MCM4, MCM5)* | Kang *et al*., 2014 |
| pSKM003 | *pRS404 (GAL1,10-MCM6, MCM7)* | Kang *et al*., 2014 |
| pSKM004 | *pRS403 (GAL1,10-MCM2, Flag-MCM3)* | Kang *et al*., 2014 |
| pALS1 | *pRS305 (GAL1,10-Cdt1, GAL4)* | Kang *et al.,* 2014 |
| pUC19-ARS1 | *ARS1* | Heller *et al.,* 2011 |
| pUC19-A-B2- | *ARS1* with A- and B2- mutations | Heller *et al.,* 2011 |
| pJF17 | *pRS304 (GAL1,10-ORC3, ORC4)* | Yeeles et al, |
| pJF18 | *pRSS303(GAL1,10-ORC3, ORC4)* | Yeeles et al, |
| pIA01 | *pRS306(GAL1,10-ORC1∆BAH-, ORC6)* | This Study |
| pET28a Abf1 2xFlag | Expression construct for Abf1 | Eaton et al |
| pIA02 | *pRS403 (GAL1,10-MCM2-2A, Flag-MCM3)* | This study |
